# Children and young adults with familial hypercholesterolaemia (FH) have healthier food choices particularly with respect to dietary fat sources compared with non-FH children

**DOI:** 10.1017/jns.2013.27

**Published:** 2013-10-11

**Authors:** Ingunn Molven, Kjetil Retterstøl, Lene F. Andersen, Marit B. Veierød, Ingunn Narverud, Leiv Ose, Arne Svilaas, Margareta Wandel, Kirsten B. Holven

**Affiliations:** 1 Department of Nutrition, Institute of Basic Medical Sciences, University of Oslo, Oslo, Norway; 2 The Lipid Clinic, Oslo University Hospital, Oslo, Norway; 3 Department of Biostatistics, Institute of Basic Medical Sciences, University of Oslo, Oslo, Norway; 4 Department of Health, Nutrition and Management, Faculty of Health Sciences, Oslo and Akershus University College of Applied Sciences, Oslo, Norway

**Keywords:** Familial hypercholesterolaemia, Children, Diet, Dietary fat sources, FH, familial hypercholesterolaemia, FH (12–14), FH subjects aged 12 to 14 years, FH (18–28), FH subjects aged 18 to 28 years

## Abstract

Familial hypercholesterolaemia (FH) leads to elevated plasma levels of LDL-cholesterol and increased risk of premature atherosclerosis. Dietary treatment is recommended to all patients with FH in combination with lipid-lowering drug therapy. Little is known about how children with FH and their parents respond to dietary advice. The aim of the present study was to characterise the dietary habits in children with FH. A total of 112 children and young adults with FH and a non-FH group of children (*n* 36) were included. The children with FH had previously received dietary counselling. The FH subjects were grouped as: 12–14 years (FH (12–14)) and 18–28 years (FH (18–28)). Dietary data were collected by SmartDiet, a short self-instructing questionnaire on diet and lifestyle where the total score forms the basis for an overall assessment of the diet. Clinical and biochemical data were retrieved from medical records. The SmartDiet scores were significantly improved in the FH (12–14) subjects compared with the non-FH subjects (SmartDiet score of 31 *v.* 28, respectively). More FH (12–14) subjects compared with non-FH children consumed low-fat milk (64 *v.* 18 %, respectively), low-fat cheese (29 *v.* 3%, respectively), used margarine with highly unsaturated fat (74 *v.* 14 %, respectively). In all, 68 % of the FH (12–14) subjects and 55 % of the non-FH children had fish for dinner twice or more per week. The FH (18–28) subjects showed the same pattern in dietary choices as the FH (12–14) children. In contrast to the choices of low-fat dietary items, 50 % of the FH (12–14) subjects consumed sweet spreads or sweet drinks twice or more per week compared with only 21 % in the non-FH group. In conclusion, ordinary out-patient dietary counselling of children with FH seems to have a long-lasting effect, as the diet of children and young adults with FH consisted of more products that are favourable with regard to the fatty acid composition of the diet.

Familial hypercholesterolaemia (FH) is a disorder usually caused by mutations in the LDL receptor gene, resulting in 2- to 3-fold elevated plasma levels of LDL-cholesterol^(^^1^^,^^2^^)^ and increased risk of premature atherosclerosis and coronary artery disease.

The European Society of Cardiology/European Atherosclerosis Society (ESC/EAS) Guidelines for the management of dyslipidaemias propose recommendations for specific nutrient composition of the diet as a part of therapeutic lifestyle changes in LDL-lowering therapy^(^^3^^)^. Dietary treatment is recommended to all patients with FH in combination with lipid-lowering drug therapy^(^^4^^)^. For children with FH, dietary recommendations are the first-line therapy, since cholesterol-lowering medication is usually not initiated before 10 to 14 years of age^(^^5^^)^. The FH patients at the Lipid Clinic are advised according to clinical guidance from the National Lipid Association Expert Panel on Familial Hypercholesterolemia^(^^4^^)^. The principles of a cholesterol-lowering diet in FH subjects include reductions in the intake of total fat, SFA and cholesterol^(^^3^^,^^4^^)^. A study by Tonstad *et al.*^(^^6^^)^ found that plasma lipids in children with FH was associated with body fatness of the child, the diet and the parental lipid profile rather than the type of FH mutation, suggesting that lifestyle is important for plasma lipids in children with FH. In non-FH patients, a total serum cholesterol level reduction of 10–30 % has been shown achievable through dietary adjustments^(^^7^^,^^8^^)^. However, little is known about the effectiveness of dietary treatment in FH subjects and only a very few studies have investigated relationships between diet and cholesterol levels in children with FH. A Cochrane review from 2010 summarised that too few studies were available to make conclusions about the effectiveness of a cholesterol-lowering diet in patients with FH^(^^9^^)^. Furthermore, little is known about how children with FH and their parents respond to dietary advice, and if and how such advice has had implications for the diet of children with FH.

The aim of the present study was therefore to describe the dietary choices in children with FH and to study if the dietary counselling provided in an ordinary out-patient clinical activity had any long-term effect on the dietary habits of the children.

## Subjects and methods

### Subjects

Subjects who had been diagnosed with FH and had attended the out-patient clinic and were aged 5–18 years at the Lipid Clinic, Oslo University Hospital in the period 2000–2010 were invited to participate (current age 5–28 years). A SmartDiet^®^ questionnaire^(^^10^^)^ and a short questionnaire to identify medication, presence of chronic disease, history of hospitalisation and possible presence of CVD in the family were filled in by the participants. The study was conducted according to the declaration of Helsinki and was approved by the Regional Committee for Medical and Health Research Ethics. Written informed consent was obtained from all participants or from one of their parents if the child was <16 years of age.

**Fig. 1.**
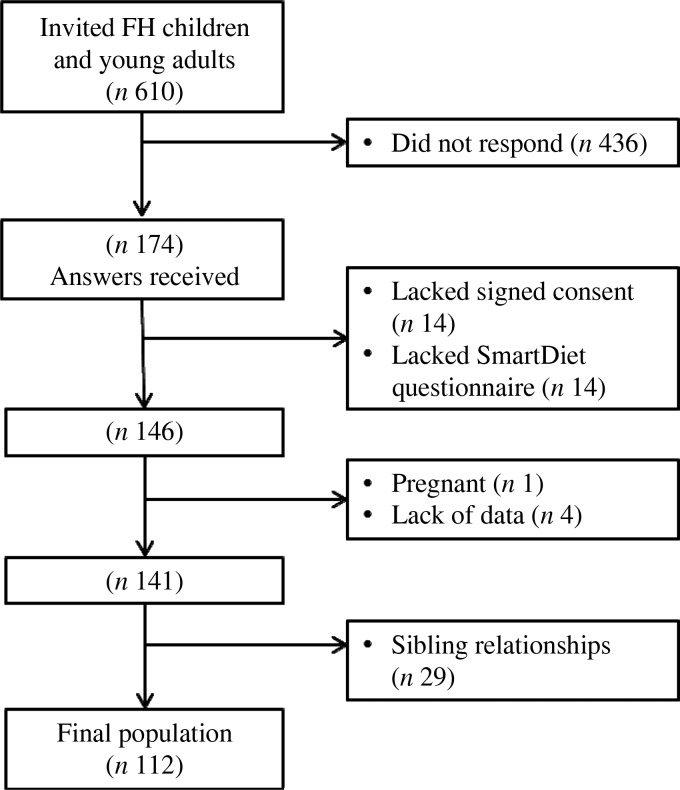
Flow chart of the study. Number of subjects invited and included and excluded in the study, and number included in the statistical analysis. FH, familial hypercholesterolaemia.

A total number of 610 patients diagnosed with FH were invited to participate in the study. Out of these, 174 responded (29 %). Some of the responses lacked either the signed informed consent or the SmartDiet questionnaire and, in total, signed informed consents and SmartDiet-questionnaires were obtained from 146 respondents (Fig. 1).

Lipid values were obtained from the medical records. Subjects who had not been evaluated with blood tests and related medical records at the Lipid Clinic during the past 4 years were not included in the study (*n* 4). Subjects who were pregnant at either the time of blood sampling or the time of food registration were also excluded (*n* 1). Among the 141 subjects who fulfilled the inclusion criteria, fifty-four had one or two siblings within the group. As sibling relationships would cause dependency among participants and possibly affect the results of statistical analysis, twenty-nine subjects among the siblings were randomly excluded. In total, 112 children and young adults with FH were included in the study sample.

A non-FH group of children was recruited from an ongoing project at the University of Oslo for comparison. A total of thirty-six children from two classes at a middle school in Oslo were invited to complete a SmartDiet questionnaire and to deliver blood samples at the Lipid Clinic. In all, twenty-nine non-FH children delivered signed consent, a completed SmartDiet questionnaire and gave a blood sample. In the non-FH group of children, one child used a vitamin A derivate, but was included in the analysis since the lipid levels were so low that any major influence on the plasma lipids was considered unlikely.

### Dietary counselling

All FH children had received dietary counselling, individually or together with the family at various occasions, at the Lipid Clinic. The lifestyle recommendations focused on: (1) reduced intakes of saturated fats and cholesterol (total fat, 25–35 % of energy intake; SFA, 7 % of energy intake; and dietary cholesterol intake, 200 mg/d); (2) use of plant stanol or sterol esters (2 g/d); (3) use of soluble fibre (10–20 g/d); (4) physical activity and energy intake to achieve and maintain a healthy body weight; (5) limitation of alcohol consumption; (6) recommendation to avoid use of any tobacco products.

### Collection of dietary data

Dietary data were collected by SmartDiet, a short self-instructing questionnaire on diet and lifestyle^(^^10^^)^. The questionnaire was developed by the Lipid Clinic, Oslo University Hospital, to easily assess diet and lifestyle habits in clinical settings and was validated in 2002 among adults^(^^10^^)^. It provided a good estimate of dietary fat and fibre, but was shown to be less accurate in terms of estimating the intake of vegetables, fish and snacks^(^^10^^)^.

The SmartDiet questionnaire consists of twenty-one questions about foods. Fourteen questions contribute to a total score. Each of these questions has three or four response categories, each giving a score of 1, 2 or 3. The total score (range 14–41) forms the basis for an overall assessment of the diet and food quality. Average use of certain groups of food is registered, either in a quantitative or qualitative way. Questions contributing to the total score that give an assessment of food quality reflect the use of milk and dairy products, cheese, meat spreads, meat for dinner, butter/margarine/oil, and bread and cereals. Questions contributing to the total score in which consumption is measured in a more quantitative way is related to the use of fish spread or fish as part of a salad, fish for dinner, mayonnaise spreads, fruits/berries/vegetables, sweet spread/sweet drinks and snacks. Intakes of fish, mayonnaise spreads and snacks are registered as weekly consumption, while in the case of vegetables, fruits and berries and sweet spreads/sweet drinks, daily consumption is registered. Additional questions include the use of products containing plant sterols, regular use of legumes, nuts/almonds, avocados, eggs, rice, potato or pasta, *n*-3 supplements and the amount of alcohol consumed per week. In cases where the participant had ticked more than one option to a question, the mean score of the ticked alternatives was used in the calculation of the total score. The exceptions were in cases where the respondent had ticked for rare use and another option in the same question. In these cases, the tick for rare use was used. Total SmartDiet score was not calculated in cases where response to one or more question was lacking. However, data may still be available from these subjects regarding dietary choices (e.g. type of milk and cheese, etc.), and these answers were included in the tables describing the dietary choices.

### Collection of clinical and biochemical characteristics

The clinical and biochemical data on the subjects with FH were retrieved from their individual patient records at the Lipid Clinic. Medical records including blood sample reports were selected so that the time gap between the blood sampling and the dietary registration was made as small as possible. Of all lipid measurements used in the present study, 75 % had been taken before the SmartDiet completion, and 25 % afterwards. Of the blood tests, 46 % were within 6 months from the SmartDiet registration, 61 % within 1 year, and only 9 % of registrations were more than 2 years apart. In all, the average time range between dietary registration and blood sampling was 45 weeks. Clinical data from medical records included statin treatment, any other use of medications, clinical manifestations of FH, information on other chronic disease and FH mutation. Biochemical parameters that were collected from the medical records included fasting concentrations of total cholesterol, LDL-cholesterol, HDL-cholesterol, apoB, apoA1, TAG, C-reactive protein and lipoprotein (a). ApoB:apoA1 ratio was calculated from the obtained values. Of the samples, 84 % of blood samples were analysed at the Department of Medical Biochemistry, Oslo University Hospital, with the rest analysed at other accredited laboratories. Blood samples from the non-FH group were all collected at the Lipid Clinic, Oslo University Hospital, and analysed at the Department of Medical Biochemistry, Oslo University Hospital. All blood samples in the non-FH group were collected within 3 weeks after the completion of the SmartDiet questionnaire. Any use of medications was recorded at the time of blood sampling.

### Statistical analysis

The results are presented as frequencies (%) for categorical variables and medians with interquartile ranges (Q1–Q3) for continuous variables. In the presentation of the results, the upper two categories of ‘fish spread’ and ‘fish for dinner’ were collapsed, cod liver oil and fish oil/*n*-3 capsules were dichotomised (uses supplements containing *n*-3; yes/no) and intakes of vegetables, fruits and berries were dichotomised (<2 portions per d, ≥2 portions per d).

We present results for all the FH subjects (age 5–28 years). However, in order to compare FH adolescents that were more similar in age range to the non-FH adolescents, we grouped FH subjects as 12–14 years (FH (12–14)) and 18–28 years (FH (18–28)). In the statistical analyses, we compared FH (12–14) with the non-FH subjects, and FH (12–14) with FH (18–28). The comparisons were performed by the Mann–Whitney *U* test or the *t* test for continuous variables depending on the distribution, and the χ^2^ test or Fisher's exact test for categorical variables depending on the expected cell frequencies. Associations between two continuous variables were estimated by Spearman's rank correlation coefficient (*r*_sp_). The level of statistical significance was set at *P* < 0·05. Statistical analyses were performed using SPSS Statistics version 18.0 or 19.0 for Windows.

## Results

### Characteristics of subjects

Selected characteristics of the subjects are shown in Table 1. The non-FH group included twenty-nine subjects, all aged 13 years. The whole FH group included 112 subjects aged 5–28 years, FH (12–14) included twenty-eight subjects and FH (18–28) included thirty-seven subjects. The FH (12–14) children had significantly higher levels of total cholesterol, LDL-C, apoB and apoB:apoA1 ratio than the non-FH children (*P* < 0·001 for all). Lipid levels of the non-FH children were within reference values. The FH (18–28) were significantly older (*P* < 0·001) and had significantly lower LDL-cholesterol (*P* = 0·02), lower apoB:apoA1 ratio (*P* = 0·006), higher apoA1 and C-reactive protein (*P* = 0·01 and *P* = 0·03, respectively) than FH (12–14). Furthermore, a larger proportion of the FH (18–28) were on statin treatment (*P* = 0·02) compared with FH (12–14).

**Table 1.** Baseline characteristics of Norwegian children with familial hypercholesterolaemia (FH) and non-FH children (Medians and interquartile ranges (IQR) or number of subjects and percentages)

	All FH subjects (*n* 112)			FH (18–28) (*n* 37)			FH (12–14) (*n* 28)			Non-FH (*n* 29)				
	*n*	Median	IQR	*n*	Median	IQR	*n*	Median	IQR	*n*	Median	IQR	P*	*P*†
Demographics														
Age on date of SmartDiet completion (years)	112	15	12–18	37	21	18–23	28	13	12–14	29	13	13–13	0·532	<0·001
Female	112			37			28			29			0·36‡	0·15‡
*n*		65			25			14			11			
%		58			68			50			38			
Drug treatment														
Statin	112			37			28							0·02
*n*		46			27			7			–		–	
%		41·1			73·0			25						
Other lipid-lowering drugs	112			37			28							0·13‡
*n*		4			4			0			–		–	
%		3·6			10·8			0·0						
Physical examination														
Xanthomas	108			34			28							
*n*		2			1			0			–		–	–
%		1·9			2·9			0·0						
Xanthelasms	107			34			28							
*n*		1			0			0			–		–	–
%		0·9			0·0			0·0						
Arcus cornea	108			34			28							
*n*		0			0			0			–		–	–
%		0·0			0·0			0·0						
Tendinittis	107			33			28							0·32§
*n*		3			2			2			–		–	
%		2·8			6·1			7·1						
Thickened heal tendons	108			32			28							
*n*		2			1			0			–		–	–
%		1·9			2·9			0·0						
Laboratory parameters														
Total cholesterol (mmol/l)	112	6·2	5·2–7·5	37	5·6	5·1–6·6	28	6·4	5·2–7·3	29	3·9	3·4–4·3	<0·001	0·09
LDL-cholesterol (mmol/l)	111	4·1	3·2–5·5	37	3·6	3·1–4·7	28	4·4	3·7–5·3	29	2·0	1·8–2·4	<0·001	0·02
HDL-cholesterol (mmol/l)	112	1·3	1·2–1·5	37	1·4	1·3–1·7	28	1·4	1·1.–1·5	29	1·4	1·3–1·6	0·13	0·1
ApoA1 (g/l)	109	1·3	1·2–1·5	35	1·4	1·3–1·6	28	1·3	1·2–1·4	29	1·3	1·3–1·4	0·1	0·01
ApoB (g/l)	109	1·0	0·9–1·3	35	0·9	09–1·1	28	1·1	1·0–1·2	29	0·5	0·5–0·6	<0·001	0·08
ApoB:apoA1	109	0·80	0·67–1·00	35	0·69	0·55–0·89	28	0·85	0·72–1·04	29	0·38	0·33–0·46	<0·001	0·006
TAG (mmol/l)	112	0·8	0·6–1·0	37	0·8	0·6–1·0	28	1·0	0·7–1·0	29	0·7	0·5–1·0	0·07	0·26
Lipoprotein (a) (mg/l)	100	263	123–514	34	263	88–409	21	225	152–512	29	100	100–247	0·016	0·05
C-reactive protein (mg/l)	101	0·6	0·6–1·0	35	0·6	0·6–1·3	22	0·6	0·6–0·6	29	0·6	0·6–0·6	0·51	0·03

FH (18–28), FH subjects aged 18–28 years; FH (12–14), FH subjects aged 12–14 years; non-FH, non-FH children (age 13 years).

* *P* values from FH (12–14) *v.* non-FH (Mann–Whitney *U* test).

† *P* values from FH (12–14) *v.* FH (18–28) (Mann–Whitney *U* test).

‡ χ^2^ Test.

§ Fisher's exact test.

### SmartDiet score

SmartDiet scores were obtained from 100 subjects with FH, thirty-three in the category FH (18–28), twenty-five in the category FH (12–14) and twenty-seven non-FH children. The median levels of the SmartDiet scores in each group are shown in Table 2. The SmartDiet scores were significantly higher in the FH (12–14) subjects than the non-FH subjects (*P* < 0·001). There was no significant difference in SmartDiet scores between FH (18–28) and FH (12–14) (*P* = 0·10; Table 2) and between FH children receiving statin treatment or not (*P* = 0·64; data not shown).

**Table 2.** SmartDiet scores in subjects with familial hypercholesterolaemia (FH) and non-FH subjects (Number of subjects, medians and interquartile ranges (IQR))

	FH (*n* 112)			FH ≥ 18 (*n* 33)			FH (12–14) (*n* 28)			Non-FH (*n* 29)				
	*n*	Median	IQR	*n*	Median	IQR	*n*	Median	IQR	*n*	Median	IQR	P*	*P*†
SmartDiet score	100	32·0	30·0–34·0	33	33·0	29·5–35·0	25	31·0	30–34	27	28·0	25·5–29·0	<0·001	0·1

FH ≥ 18, FH subjects aged 18 years or older; FH (12–14), FH children aged 12–14 years; non-FH, non-FH children (age 13 years).

* *P* value from SmartDiet scores of FH children aged 12 to 14 years *v.* non-FH children (tested with Mann–Whitney *U* test).

† *P* value from SmartDiet scores of FH children aged 12 to 14 years *v.* FH subjects aged 18 years or older (tested with Mann–Whitney *U* test).

### Food choices

Tables 3 and 4 show which food items among the different food categories that were most frequently consumed. Significant differences between the FH (12–14) and the non-FH children were observed regarding type of milk (*P* < 0·001), cheese (*P* = 0·005) and types of meat for dinner (*P* = 0·016) (Table 3). The same pattern was observed among the older FH (18–28) as for the FH (12–14) subjects (Table 3). We found no significant difference regarding egg consumption between the FH (12–14) and non-FH subjects and between the FH (12–14) and FH (18–28) subjects.

**Table 3.** Frequency table of food items among categories of foods that are chosen most frequently in subjects with familial hypercholesterolaemia (FH) and non-FH subjects (Number of subjects and percentages or medians and interquartile ranges)

	FH													
	All FH subjects (*n* 112)			FH ≥ 18 (*n* 37)			FH (12–14) (*n* 28)			Non-FH (*n* 29)				
	*n*	*n*	%	*n*	*n*	%	*n*	*n*	%	*n*	*n*	%	P*	*P*†
Milk	108			37			28			28			<0·001	0·52
≤1 litre per week		10	9·3		3	8·1		5	17·9		4	14·3		
Whole fat		0	0		0	0·0		0	0·0		2	7·1		
Low-fat milk		26	24·1		8	21·6		5	17·9		17	60·7		
Skimmed		72	66·7		26	70·3		18	64·3		5	17·9		
Cheese	103			35			24			29			0·005	0·33
≤ Once per week		11	10·7		6	17·1		1	4·2		3	10·3		
Whole-fat cheese		29	27·2		10	28·6		9	37·5		22	75·9		
Medium-fat cheese		35	34·0		13	37·1		7	29·2		3	10·3		
Low-fat cheese		29	28·2		6	17·1		7	29·2		1	3·4		
Meat as cold cuts	107			36			26			28			0·24	0·67
≤ Once per week		13	12·1		4	11·1		3	11·5		4	14·3		
Fat meat		17	15·9		5	13·9		6	23·1		12	42·9		
Lean meat (<10 % fat)§		77	72		27	75·0		17	65·4		12	42·9		
Meat for dinner	107			37			26			29			0·016	0·47
≤ Once per week		3	2·8		1	2·7		0	0·0		1	3·4		
High-fat cuts		3	2·8		3	8·1		0	0·0		7	24·1		
Medium-fat cuts		10	9·3		5	13·5		3	11·5		4	13·8		
Lean cuts		91	85		28	75·7		23	88·5		17	58·6		
Eggs per week	107			34			28			27			0·32‡	0·72‡
Median		2			2			2			2			
Interquartile range		1·0–2·25			1·0–2·0			1·0–2·5			1·5–3·0			
Butter and margarine on bread	110			37			27			29			<0·001	0·054
Usually no butter or margarine		28	25·5		15	40·5		5	18·5		7	24·1		
Butter/hard margarine		3	2·7		1	2·7		0	0·0		13	44·8		
Soft margarine		4	3·6		0	0·0		2	7·4		5	17·2		
Margarine with highly unsaturated fat		75	68·2		21	56·8		20	74·1		4	13·8		

FH ≥ 18, FH subjects aged 18 years or older; FH (age 12–14), FH subjects aged 12 to 14 years, non-FH, non-FH children (age 13 years).

* *P* values from FH (12 to 14) *v.* non-FH children (Fisher's exact test).

† *P* values from FH children (12 to 14) *v.* FH subjects (≥18) (Fisher's exact test).

‡ Mann–Whitney *U* test.

§ Oil-based pâtés in this category contain about 20 % fat, but are highly unsaturated.

**Table 4.** Frequency table of food items among categories of foods that are chosen most frequently in subjects with familial hypercholesterolaemia (FH) and non-FH subjects (Number of subjects and percentages)

	FH													
	All FH subjects (*n* 112)			FH ≥ 18 (*n* 37)			FH (12–14) (*n* 28)			Non-FH (*n* 29)				
	*n*	*n*	%	*n*	*n*	%	*n*	*n*	%	*n*	*n*	%	P*	*P*†
Fish on bread	109			36			28			28			0·31	0·07
One slice per week or less		68	62·4		19	52·8		21	75		24	85·7		
On two or more slices per week		41	37·6		17	47·2		7	25		4	14·3		
														
Fish for dinner	111			37			28			29			0·33	0·36
Once per week or less		41	36·9		16	43·2		9	32·1		13	44·8		
Two times per week or more		70	63·1		21	56·8		19	67·9		16	55·2		
														
*n*-3 Supplements	109			36			27			25			0·81	0·61
Does not use supplement containing *n*-3		46	42·2		17	47·2		11	40·7		11	44·0		
Uses supplement containing *n*-3		63	57·8		19	52·8		16	59·3		14	56·0		
														
Bread type/grain products	108			37			26			28			0·4‡	0·067‡
Do not eat bread/grain products		2	1·9		0	0·0		1	3·8		0	0·0		
Low in fibre		30	27·8		8	21·6		11	42·3		9	32·1		
High in fibre		76	70·4		29	78·4		14	53·8		19	67·9		
														
Vegetables/fruits/berries	106			36			28			29			0·33	0·92
Less than two portions per d		38	35·8		12	33·3		9	32·1		13	44·8		
Two or more portions per d		68	64·2		24	66·7		19	67·9		16	55·2		
														
Plant sterols	105			36			27			27			<0·001	0·63
Does not use a product containing plant sterols		58	55·2		24	66·7		13	48·1		26	96·3		
Uses a product containing plant sterols		47	44·8		12	33·3		14	51·9		1	3·7		
														
Sweet spreads or sweet drinks	108			37			26			28			0·03	0·1
Less than two times per d		72	66·7		26	70·3		13	50·0		22	78·6		
Two or more times per d		36	33·3		11	29·7		13	50·0		6	21·4		
														
Snacks	109			37			26			29			0·06	0·72
Less than two times per week		38	34·9		13	35·1		8	30·8		3	10·3		
Two or more times per week		71	65·1		24	64·9		18	69·2		26	89·7		

FH ≥ 18, FH subjects aged 18 years or older; FH (age 12–14), FH subjects aged 12 to 14 years, non-FH, non-FH children (age 13 years).

* *P* values from FH (12 to 14) *v.* non-FH children (χ^2^ test).

† *P* values from FH children (12 to 14) *v.* FH subjects (≥18) (χ^2^ test).

‡ Fisher's exact test.

Regarding choice of margarine, significant differences were also observed between FH (12–14) and non-FH subjects and between FH (12–14) and FH (18–28). Of the subjects, 74 % of the FH (12–14) children and only 14 % of the non-FH children reported that they used margarine with a high proportion of unsaturated fatty acids on bread (*P* < 0·001). Furthermore, a larger percentage of the older FH subjects (41 %) were not using any margarine compared with the younger FH subjects (19 %) (*P* = 0·054).

No significant differences were observed between the FH (12–14) and the non-FH group with regard to the use of fish spread on bread, fish for dinner, use of *n*-3 supplements, types of bread and grain products used or portions of fruit and vegetables per d (0·31 ≤ *P* ≤ 0·81) (Table 4). However, 78 % of the older FH subjects (18–28) chose bread with high fibre content compared with 53 % in the FH (12–14) group (*P* = 0·067).

The use of plant sterol-containing products differed significantly between the FH (12–14) and the non-FH group (*P* < 0·001) (Table 4).

There was a significant difference in the use of sweet spreads and drinks (*P* = 0·03), where only 21 % of the non-FH children reported use of sweet spreads and drinks more than twice daily, in contrast to FH (12–14) where 50 % reported the same. The older FH subjects (18–28) had a similar pattern with respect to use of sweet spreads as the non-FH children. In contrast, the opposite pattern was seen regarding use of snacks where 69 % of the FH (12–14) reported intake more than twice per week in contrast to the non-FH children, where 89 % reported the same (*P* = 0·06).

### Lipid levels and SmartDiet scores

No significant correlations were observed between SmartDiet scores, blood lipid levels and C-reactive protein in the total group of FH subjects (–0·1 ≤ *r*_sp_ ≤ 0·09), FH (18–28) (–0·07 ≤ *r*_sp_ ≤ 0·20) or FH (12–14) (–0·054 ≤ *r*_sp_ ≤ 0·029). To investigate if statin treatment had any effect, correlations were performed separately in statin-treated (*n* 43) and non-statin-treated (*n* 57) FH subjects. However, no significant correlations were observed in the FH (12–14) or FH (18–28) groups (–0·479 ≤ *r*_sp_ ≤ 0·537 and –0·1 ≤ *r*_sp_ ≤ 0·19, respectively), although plasma TAG tended to be moderately inversely correlated with the SmartDiet score in the statin-treated FH (12–14) group (*r*_sp_ –0·479; *P* = 0·071; *n* 15). In the non-FH group (*n* 27), however, TAG level and SmartDiet score were moderately inversely correlated (*r*_sp_ –0·38; *P* = 0·05).

## Discussion

The present study found that children with FH had healthier food choices, particularly with respect to the most important dietary fat sources for saturated fat.

Observations of dietary consistency from adolescence into adulthood have been found in other studies, supporting the beneficial role of implementing healthy dietary habits at an early age, and targeting nutrition education especially at children and adolescents^(^^11^^)^. The SmartDiet scores of the FH (18–28) were in line with those of the younger FH subjects. It is therefore tempting to speculate that the dietary habits achieved in childhood, at least with regard to low-fat food choices, seem to last into early adulthood. Although the FH (12–14) subjects had better SmartDiet scores than the non-FH children, they still had potential for improvement of the diet.

The analysis of frequency of food items used within the different food categories in the SmartDiet questionnaire indicated that the higher SmartDiet scores of the FH children aged 12–14 years were based on a systematic pattern of choosing low-fat alternatives, or alternatives high in unsaturated fats. The Norwegian nationwide survey on dietary habits among Norwegian 4th graders (9 years old) and 8th graders (13 years old)^(^^12^^)^ found that meat, dairy products, butter, margarine and oil were the most important sources of fat in the children's diets, contributing with approximately 50 % of dietary fat^(^^12^^)^. The FH children and young adults appear to choose low-fat and highly unsaturated fat alternatives among these foods. Thus, it appears that they are choosing favourable alternatives among the foods where there is the most to gain in food quality. The use of high-fat or medium-fat content products was more widespread among the non-FH children participating in the present study in accordance with results from a previous nationwide study^(^^12^^)^.

The FH (12–14) and the non-FH children did not differ significantly in either fish consumption, consumption of vegetables, berries and fruit or in use of fibre-rich grain products. This may suggest that the FH children are more responsive to dietary advices regarding fat intake than those that might appear less related to fat and cholesterol. However, the use of both fibre-rich bread (54 and 68 % in FH children (12–14) and non-FH children, respectively) and consumption of fruit, vegetables and berries more than twice per week (68 and 55 % in FH children (12–14) and non-FH children, respectively) was very widespread among both groups of children. There was a similar pattern with respect to the use of *n*-3 dietary supplements among FH children aged 12–14 years and the non-FH children where 59 and 56 %, respectively, reported regular use of *n*-3 dietary supplements.

The FH (12–14) children had a healthier diet than the non-FH group regarding most of the food choices; however, the FH children used sweet spreads and sweet drinks significantly more often than the non-FH children. FH children may have higher intakes of sweet spreads such as jam and honey, as well as sugar drinks, reflecting that the health focus in FH is on saturated fat rather than on sweets.

However, in dietary studies, one can never overlook the problem of selective misreporting. The general nutritional advice in Norway is to cut down on both fat and sugar-rich foods, whereas the advice to the FH children was focused more specifically on fat-rich foods. Thus, a pleasing bias that would affect the reporting of intake of fat-rich foods, more than sugar-rich foods for the FH children, cannot be ruled out. In the present study, however, the dietary registration was not performed under supervision of the Lipid Clinic, but was performed at home, and thereafter the questionnaire was mailed directly to the project leader, potentially minimising the pleasing bias. Furthermore, we have previously shown that patients with genetically verified FH had a more favourable diet than patients with lifestyle-induced hypercholesterolaemia, where both groups had received the same dietary advice. This was primarily due to food choices of low-fat milk, cheese and meat, similar to the food choices reported for the FH children. It seemed that genetic confirmation of hypercholesterolaemia helped to increase the motivation for a more healthy diet^(^^13^^)^.

We found no significant correlations between the SmartDiet scores and blood lipid levels in FH (12–14) subjects or in the total group of FH subjects. It is likely that the sensitivity of the rather simple dietary questionnaire SmartDiet was too low to provide such information when the number of subjects is relatively limited. The questionnaire is designed to give an overall score of the diet, where the score of different fat sources such as dairy products, meat and margarine contribute to the total score in a similar way as the scores for intake of non-fat-contributing food items such as fruit and vegetable intake, fibre-rich cereals and consumption of sweet spreads or sweet drinks. Furthermore, the fact that the SmartDiet questionnaire and the blood samples were not collected simultaneously may also influence the results. The average time between the two measurements was 45 weeks in the present study. This is a limitation of the study. However, studies have found that dietary patterns and food choices are fairly stable in the time between adolescence and adulthood^(^^11^^)^ and from the 4th to 7th grade^(^^14^^)^ although adolescence may be a period when individuals achieve new dietary habits as well. With respect to blood lipids, a recent study of 10 years of consecutive blood lipid patterns in normocholesterolaemic children aged 8 to 18 years demonstrated only small variations in total cholesterol, LDL-cholesterol, HDL-cholesterol and TAG levels with age^(^^15^^)^. In line with our findings, Tonstad *et al.*^(^^6^^)^ did not find any correlations between lipid levels in FH children and dietary intakes when the dietary intake was measured by a 4-d dietary record.

Other limitations of the study are that only about 30 % of the invited FH patients responded to the invitation, which may have introduced selection bias in the sample, and the relatively small non-FH group that was used as a reference group. Whereas the FH subjects were recruited from the whole country, with the majority coming from the southeastern part of Norway, the non-FH children were recruited exclusively from a high socio-economic district in Oslo. However, it has been reported that children of high-income parents have healthier dietary habits than children from low-income homes^(^^16^^)^, thus, if anything, the observed difference in dietary score between the FH and the non-FH children is more likely to be underestimated than overestimated, underscoring the improved quality of the diet in children with FH.

In conclusion, children and young adults with FH had a healthier diet than non-FH children, in particular with respect to low-fat products and products that are favourable with regard to the fatty acid composition of the diet. However, we observed a higher intake of sugar-rich foods compared with non-FH children. This suggests that dietary awareness initiated early in childhood may lead to a long-term improved dietary quality in children and young adults with FH, except for the intake of sugar-rich foods.
